# The Effect of Culture Mix or Not Conflict Against the Decision Making—A fNIRS Study

**DOI:** 10.1002/ijop.70253

**Published:** 2026-07-24

**Authors:** Yanping He, Yutong Ma, Wenhao Lv, Haiqi Xiang, Xiangyu Yan, Yinfeng Tan, Fangfang Liu, Lu Gan, Yelan Zhang, Caiqing Chen, Peiyao Liu, Shuang Wei, Fan Xu, Hong Shi

**Affiliations:** ^1^ Tourism School Southwest Minzu University Chengdu Sichuan People's Republic of China; ^2^ Department of Evidence Based Medicine and Social Medicine, School of Public Health Chengdu Medical College Chengdu Sichuan People's Republic of China; ^3^ Sichuan Provincial Key Laboratory of Philosophy and Social Sciences for Intelligent Medical Care and Elderly Health Management Chengdu Medical College Chengdu Sichuan People's Republic of China; ^4^ Art College, Southwest Minzu University Sichuan China

**Keywords:** cognitive conflict, decision making, facial expressions, fNIRS, vital signs

## Abstract

With increasing globalisation and deepening integration of local cultures, a growing number of product advertisements incorporate diverse, mixed cultural elements, designed to attract consumer attention and enhance purchase interest. However, the underlying mechanisms by which visual stimuli successfully influence consumer purchase behaviour remain poorly understood. In the present study, 300 college students aged 18–22 were recruited and randomly assigned to one of three between‐subjects experiments (A, B and C), each comparing a control group (*n* = 50) with an experimental group (*n* = 50). The experiment employed a single‐exposure paradigm, using functional near‐infrared spectroscopy (fNIRS) to monitor changes in participant haemoglobin in brain cortical areas. Facial expressions and vital signs were simultaneously recorded in parallel. Based on these measurements, visual stimuli with mixed cultural elements collectively influence consumer decision‐making through their intensity characteristics, potentially involving cognitive conflict processing and may be associated with enhanced emotional arousal and modulated autonomic nervous activity. These results provide exploratory evidence for the neural correlates underlying visual strategies in cross‐cultural marketing and offer new methodological support for brand design and consumer cognition studies.

**Abbreviations**
DBP: diastolic blood pressure
fNIRS: functional near‐infrared spectroscopy
HBO: haemoglobin oxygen
SBP: systolic blood pressure
SDNN: standard deviation of normal‐to‐normal heartbeat
SPO_2_
: peripheral capillary oxygen saturation

## 1 Introduction

With increasing cultural globalisation and integration, the commercial market for mixed‐culture products is trending toward exponential growth. While this phenomenon may at first appear to be merely due to supply side innovations, numerous young adults have grown up against a backdrop of increasing cultural confidence and are embracing the convergence of diverse cultural elements while maintaining a strong sense of identity and belonging rooted in their native cultures (van Eijck and Lievens 2008; Arnett 2002; Jensen et al. 2011). These dual characteristics represent a key driving force behind the purchase of mixed culture products by consumers (Hong et al. 2000). From a cognitive perspective, culture serves as an invisible framework that shapes the human mind and influences behaviour (Han and Northoff 2008). Through incorporation of commonly recognised symbols, cultural elements can be used to instil social values into consumer products, thereby fostering certain consumer behaviours and sustaining a stable market structure (Arnould and Thompson 2005). In contrast, culture can also constrain unconventional or inappropriate consumer purchases through establishment of cultural norms and moral boundaries (Thornton et al. 2012).

Mixed culture refers to the phenomenon where elements and symbols from diverse cultural systems come into contact, interact and clash, ultimately leading to new cultural forms (Bentley 1993). Historically, cross‐cultural contacts and interactions have been associated with historical events, from ancient trans‐regional trade activities such as the Silk Road (Trivellato et al. 2014), to the indigenisation process in religious dissemination, and further to the cultural hybridisation phenomenon spawned by colonial expansion (Bhabha 1994). Thus, events shaping mixed culture have left traces throughout history. However, with the acceleration of globalisation and the rapid advancement of digital technologies and the internet, the development of mixed culture is no longer dependent on historically spontaneous events, but rather has become a global, highly conscious and dynamically sustained consumer‐driven process with widespread participation (Canclini and Rodriguez 1995; Ihlebk 2017; Carbonell 2007).

In the present study, we hypothesise that exposure to mixed cultural stimuli may trigger cognitive conflicts by challenging participants' established cultural schema, whereas resolution of such conflicts is subject to an acceptable threshold. In the context of this hypothesis, the process of conflict resolution is critical: successful conflict resolution through cognitive adjustment and cultural learning leads to product acceptance and identification by consumers, whereas failure reinforces cultural boundaries and results in product rejection.

Young adults are the primary consumers of mixed culture; their preferences directly affect the market (Kjeldgaard and Askegaard 2006). However, current studies primarily rely on self‐reported data and thus fail to account for young adults' expressive restraint and their tendency to conceal their real preferences due to fears of aesthetic judgement and social labelling. Thus, results can be biased because study participants frequently conceal their true preferences and often choose answers in line with social expectations (Berger and Ward 2010; Marwick and boyd 2011). Under such conditions, traditional study methods suffer from systematic bias, which may lead to potentially unreliable results based on traditional surveys. In addition, decision making depends on subjective evaluation; however, the mechanisms driving consumer behaviour from visual stimulation to decision making remain unclear.

To overcome these limitations, the present study employed a multi‐modal experimental design, involving 300 college students aged 18–22, who were randomly assigned to one of three independent experiments (A, B and C), each utilising a between‐subjects design with a control group (exposed to conventional images) and an experimental group (exposed to mixed‐cultural images) (*n* = 50 per group). A 48‐channel fNIRS system was used to capture functional signals in the brains of study participants, while facial expressions and vital signs data were recorded in parallel by video recording equipment. This combined data was used to systematically measure the neurophysiological response of young adults to stimulation with mixed‐culture images.

## 2 Methods

### 2.1 Ethics Statement

This study was previously approved by an institutional ethics committee (2023 NO. 113), and all measurement processes were non‐invasive and non‐contact. Experienced staff monitored the entire process to ensure that all measurements were collected efficiently and without problems.

### 2.2 Participants

Three‐hundred healthy college students were invited to participate in the present study, including 150 males and 150 females, aged 18–22 years, with an average age of 19.4 years. Participants were randomly assigned to one of three independent experiments (Experiments A, B and C; *n* = 100 per experiment). Each experiment employed a single‐factor between‐subjects design, wherein participants were further randomly assigned to either a control condition (*n* = 50; exposed to conventional images) or an experimental condition (*n* = 50; exposed to mixed‐cultural images). Additional study inclusion criteria were as follows: individuals could not be involved in another fNIRS experiment; must have agreed to allow recording of physiological data; and must have been free of mental or other psychological disorders. Exclusion criteria were refusal to participate in the present study.

### 2.3 Priority Stimuli Validation

A prior test was conducted with 66 college undergraduate students to validate the effectiveness of the mixed‐cultural stimulation. Participants rated all images on a seven‐point Likert scale measuring perceived cultural mixing. Paired‐samples *t*‐tests revealed that the mixed‐cultural images were perceived as significantly more culturally mixed than the conventional images across all three experiments. Specifically, in experiment A, the experimental image (*M* = 4.73, SD = 1.79) presented a higher score than the control image (*M* = 3.91, SD = 1.63), *t* = −2.87, df = 65, *p* = 0.006; in experiment B, the experimental image (*M* = 4.59, SD = 1.86) presented a higher score than the control image (M = 3.38, SD = 1.86), *t* = −5.47, df = 65, *p* < 0.001; and in experiment C, the experimental image (*M* = 4.59, SD = 2.02) presented a higher score than the control image (*M* = 2.52, SD = 1.73), *t* = −8.20, df = 65, *p* < 0.001.

Additionally, all experimental images were quantitatively analysed via brightness, colour saturation, visual complexity and image entropy using Python 3.11 with OpenCV and scikit‐image. Independent‐samples Welch *t*‐tests confirmed no significant differences between the control and experimental groups (all *p* > 0.05; see Table S1). These results confirmed that our stimulus materials successfully manipulated the intended construct.

### 2.4 General Workflow

Prior to the experiment, all study participants were introduced to the general experimental procedures and safety precautions. Video recording equipment was set up to capture fine changes in facial expressions, and functional near‐infrared spectroscopy (fNIRS) was set up to record dynamic changes in haemoglobin in specific brain areas. Then, study participants were asked to sit quietly and rest for 60 s in a controlled laboratory environment, in order to establish stable, baseline physiological conditions. Once the study participants were ready, both the fNIRS system and the video recording equipment were activated simultaneously.

**FIGURE 1:**
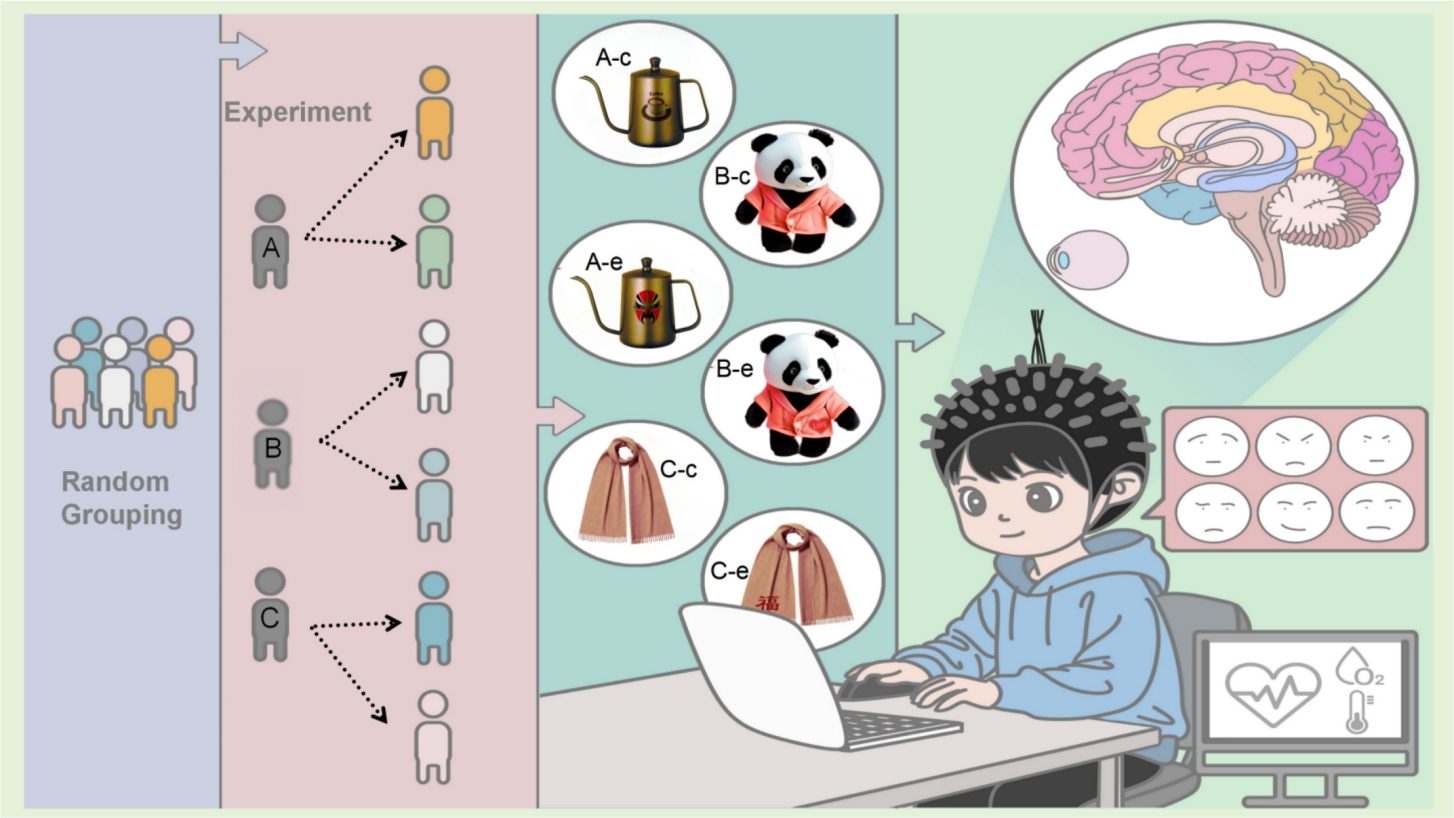
Task demonstration. The study included 300 college students (aged 18–22), who were randomly divided into one of three independent experiments (Experiments A, B and C; *n* = 100 per experiment). Each experiment employed a single‐factor between‐subjects design, wherein participants were further randomly assigned to either a control condition (A‐c/B‐c/C‐c; *n* = 50 exposed to conventional images) or an experimental condition (A‐e/B‐e/C‐e; *n* = 50 exposed to mixed‐cultural images). During exposure to images, an fNIRS system was used to capture functional signals in the brain, while video recording equipment was used to record facial expressions and vital signs data. A‐c: Experiment A control group; A‐e: Experiment A experimental group; B‐c: Experiment B control group; B‐e: Experiment B experimental group; C‐c: Experiment C control group; C‐e: Experiment C experimental group.

The whole experiment consisted of seven segments, where the duration of each segment ranged from 15 to 120 s, depending on the task requirement. The process was as follows: participants were allotted 20 s for reading each of two passages separately. Then they were required to look at an image, to understand the corresponding text materials, and choose answers to questions 1 through 3 on a paper. Next, the participants needed to complete a questionnaire‐based survey on their mobile phones. Finally, at the end of the experiment, research staff collected data to evaluate the task completion rate (for details see Figure 1).

### 2.5 Measurement of Facial Expressions and Vital Signs

Changes in facial expressions were analysed using Face Reader 6.0 (NOLDUS). Simultaneously, vital signs, including: heart rate, percutaneous arterial oxygen saturation (SPO_2_), standard deviation of normal‐to‐normal heartbeat (SDNN), systolic blood pressure (SBP) and diastolic blood pressure (DBP), were measured using a Mindray vital signs monitoring system (VS‐600).

### 2.6 fNIRS Data Acquisition

**FIGURE 2:**
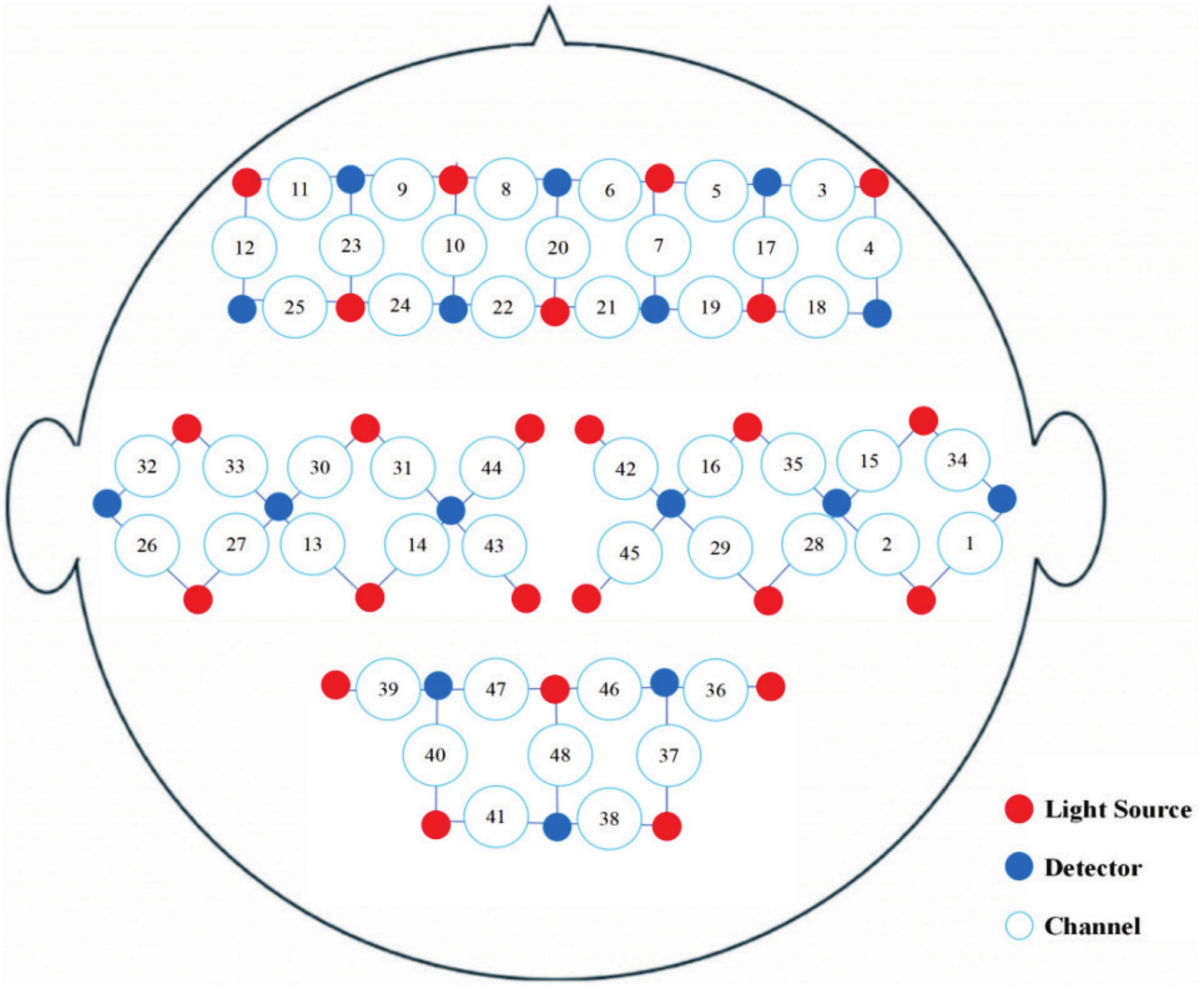
fNIRS schematic layout.

Study participants wore an fNIRS device (NirSmartII‐3000A, Danyang Huichuang Medical Equipment Co. Ltd., China) attached to an adjustable head cap. fNIRS measurements were conducted at two wavelengths (730 and 850 nm), and at a sampling rate of 11 Hz. The fNIRS system was configured with 15 sources and 16 detectors, with a source detector separation of 3 cm, resulting in 48 measurement channels. The fNIRS measurement system was designed to provide full cortical coverage, including of the frontal, bilateral temporal, parietal and occipital lobes, in order to acquire hemodynamic signals. Source‐detector positions and five points (Nz, Cz, Al, Ar, Iz) referring to the 10–20 international EEG system were mapped using an electromagnetic 3D digitiser (Patriot, Polhemus, USA, see Figure 2). All collected data were converted into MNI coordinates and further projected onto an MNI standard brain template via the spatial registration function included in NirSpace software (Danyang Huichuang Medical Equipment Co. Ltd., China). For further information on anatomical regions corresponding to each channel and their respective coverage percentages, please refer to Table S2.

### 2.7 fNIRS Data Pre‐Processing

Recorded fNIRS signals were pre‐processed using NirSpark V1.8.1 (Danyang Huichuang Medical Instrument Co. Ltd.). Specifically, head motion correction was performed first, followed by digital bandpass filtering of the raw optical density signals within the 0.01–0.2 Hz range. The selection of this band‐pass filter was specifically chosen based on our experimental design, which required the synchronous acquisition of fNIRS data alongside continuous life‐sign monitoring, including HR, SPO_2_, SDNN, DBP and SBP. In multimodal physiological recording, it is critical that the raw fNIRS signal retains the relevant frequency bands corresponding to systemic physiological activities so that these components can be accurately mapped to the synchronised life‐sign traces and subsequently regressed out. Next, the relative concentration curves of oxygenated haemoglobin (HBO), deoxygenated haemoglobin and total haemoglobin were generated via filtering. Haemo‐based conversion was then applied to derive haemodynamic concentrations from raw optical signals. Finally, after pre‐processing, channel data integrity was checked to ensure that no data was missing. During data analysis, pseudo‐differential correction was used to reduce the effect of head movement on the data. Signal conversion was based on the Beer–Lambert law, which was used to convert light absorption data to changes in the concentration of HBO. Functional connectivity strength was assessed by calculating the Pearson correlation coefficient between each pair of fNIRS channels. The fNIRS signals were then constructed into a functional connectivity network, and after obtaining the functional connectivity data, statistical analyses and visualisations were performed, specifically including between‐group comparisons, correlation analyses and brain network visualisation.

### 2.8 Statistical Analysis

All data were stored and managed in Microsoft 365, and measurement data were presented as Mean ± SD. Brain functional connectivity was calculated by Pearson correlation coefficients between the time series of the different channels to construct functional connectivity matrices for each subject during the task period. Using a statistical analysis plug‐in included in the NirSpark software suite, one‐way ANOVA was applied first to check for differences among the groups. Then, multiple comparisons were performed to determine the specific between‐group differences. fNIRS data from the six groups were compared via a one‐way ANOVA. Stata 18.0 software was used for statistical analysis, while vital signs and facial expression data were compared via a double‐sided Student's test if the data were normally distributed. Otherwise, a non‐parameter method was used. A *p* value less than 0.05 was deemed statistically significant.

## 3 Results

### 3.1 Demographics

The study sample consisted of 300 college students from 10 different ethnic groups. Study participants were randomly divided into the experimental conditions, with a male‐to‐female ratio of 1:1 maintained across conditions. No significant distribution difference was found for age and sex between the six groups (details see Table 1).

**TABLE 1:** Demographic background of all participants.

Group	A‐c	A‐e	B‐c	B‐e	C‐c	C‐e
Female	25	25	25	25	25	25
Male	25	25	25	25	25	25
Age	18.94	19.34	20	19.16	19.14	20
Ethnic	5	3	4	5	2	3

*Note:* A‐c: Experiment A control group; A‐e: Experiment A experimental group; B‐c: Experiment B control group; B‐e: Experiment B experimental group; C‐c: Experiment C control group; C‐e: Experiment C experimental group.

### 3.2 Purchase Intention

To test the effect of mixed culture on purchase intention, three independent experiments were conducted, each comparing a control group and an experimental group. Independent samples *t*‐tests were performed for each experiment. Specifically, in Experiment A (coffee pot), no significant difference was found between the control and experimental groups (*t* = −0.14, df = 98, *p* = 0.886). Besides, in Experiment B (panda), the experimental group presented marginally lower purchase intention than the control group (*t* = 1.66, df = 98, *p* = 0.099). Furthermore, in Experiment C (scarf), a significant effect was found that the control group presented the significantly higher purchase intention than the experimental group (*t* = 2.34, df = 98, *p* = 0.021). Details, please refer to Table S3.

### 3.3 fNIRS—Dynamic Haemoglobin Oxygen Changes for Compared Control and Experimental Groups

**FIGURE 3:**
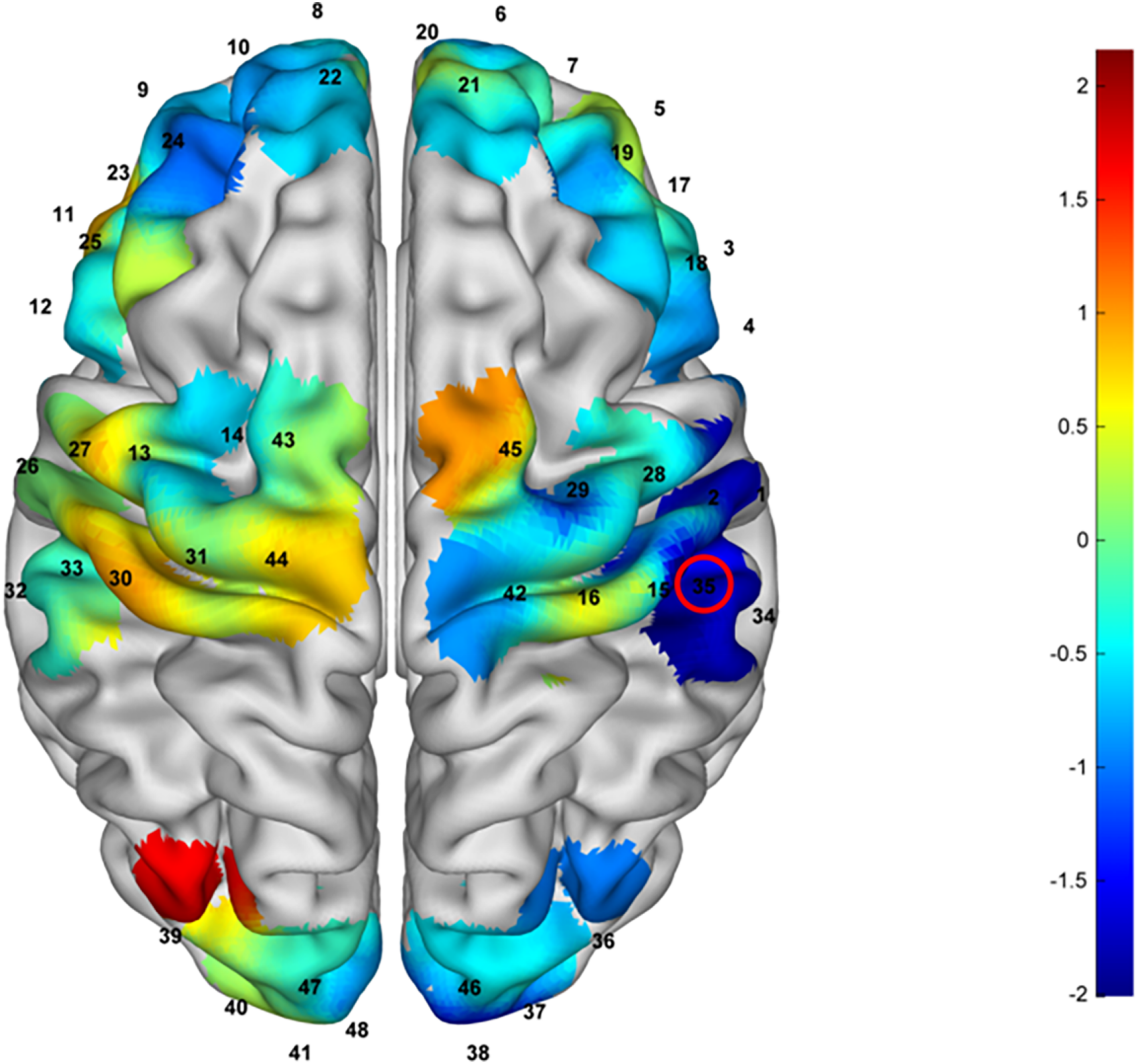
Comparison of HBO levels between matched pairs of experimental and control groups. Darker red represents higher activation intensity, lighter colours correspond to weaker cortical activation. Circle highlight area showing significant differences between the two groups.

To compare mean HBO levels between experimental groups and controls, activation maps were drawn as a function of time. The maps are presented using a colour gradient, where darker red signifies a significant degree of activation, while lighter colours indicated reduced activation (for details see Figure 3). A two‐sample *t*‐test was performed to compare mean HBO levels between compared groups. The data indicated a significant difference in CH35, the supramarginal gyrus part of Wernicke's area (*t* = −2.6408, df = 298, *p* = 0.009) (for details please see Table 2).

**TABLE 2:** Analysis of differences in mean HBO (experimental vs. control).

CH	S‐D	*T*	Degree of freedom	*p*	Significant
35	40—Supramarginal gyrus part of Wernicke's area	−2.6408	298	0.009	Yes

### 3.4 Inter‐Group Analysis of Varying Functional Connectivity

Based on prior consumer neuroscience studies on brand decision‐making (Deppe et al. 2005) and models of cognitive control and conflict monitoring (Kerns et al. 2004), we defined three regions of interest (ROIs): the dorsolateral prefrontal cortex (DLPFC), the right supramarginal gyrus (SMG‐R) and the frontopolar area (FPA). The DLPFC is centrally involved in cognitive control and conflict resolution, and the SMG‐R helps shift attention and adapt to social cues, while the FPA supports higher‐order control processes (Badre and Wagner 2004). Accordingly, we restricted our hypothesis‐driven analyses to the functional connectivity among these three ROIs.

In Experiments A (Coffee pot) and B (Panda), no significant group differences were observed for any ROI pairs (all *p* > 0.05). Furthermore, in Experiment C (Scarf), the control group showed a trend‐level difference in functional connectivity between the SMG‐R and the FPA compared to the experimental group (*t* = −2.21, df = 96.63, *p* = 0.030). However, this effect failed to pass the FDR correction for the three a priori ROI pairs tested. No significant group differences were observed for the other ROI pairs in Experiment C (all *p* > 0.05). Details see Table S4.

### 3.5 Visualisation of Functional Connectivity Between Match Groups During the Task Period

**FIGURE 4:**
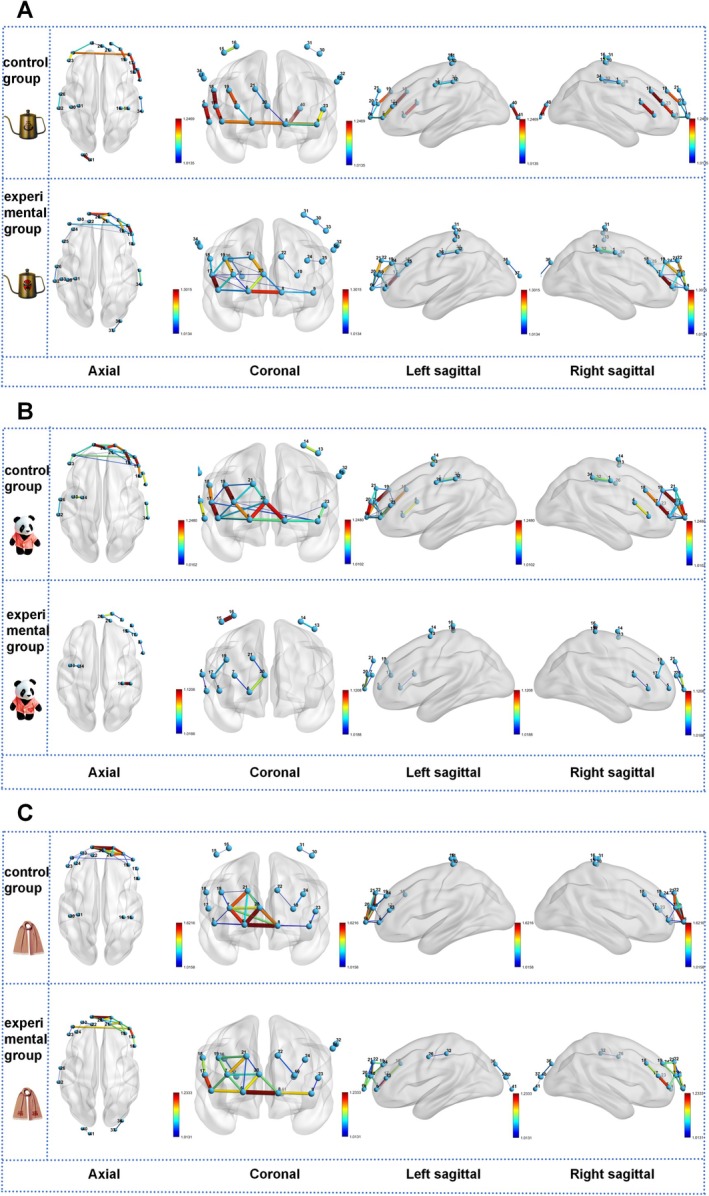
Visualisation of functional connectivity between compared groups across different brain anatomical perspectives. (A) represents the functional connectivity between the experimental and control subgroups under Experiment A (Coffee pot). (B) represents the functional connectivity between the experimental and control subgroups under Experiment B (Panda). (C) represents the functional connectivity between the experimental and control subgroups under Experiment C (Scarf).

While a task was being performed, visualisation of functional connectivity was conducted with respect to the variation strength across the different groups. In Experimental A (Coffee pot), the control group exhibited stronger connectivity in Premotor and Supplementary Motor Cortex (PSMC), DLPFC and Primary Somatosensory Cortex (PSC) (Figure 4A). While the control group displayed more heightened connectivity in PSC and Pars triangularis Broca's area (PTBA) in Experiment B (Panda) (Figure 4B). Moreover, in Experiment C (Scarf), the experimental group displayed increased connectivity in the inferior prefrontal gyrus (IPG), FPA, Supramarginal gyrus of Wernicke's area (SMG) and DLPFC (Figure 4C).

Significant differences in brain activation patterns were observed between the two conditions, indicating that distinct stimulations caused a divergence in neural processing pathways. The control groups primarily relied on experience‐driven, top‐down processing, enabling rapid access to pre‐existing internal templates for perceptual matching. In contrast, for the experimental groups, the presence of mixed cultural images induced conflict, activating an indirect compensatory pathway that demands greater cognitive resources (Benet‐Martínez et al. 2002).

### 3.6 Facial Expressions

**FIGURE 5:**
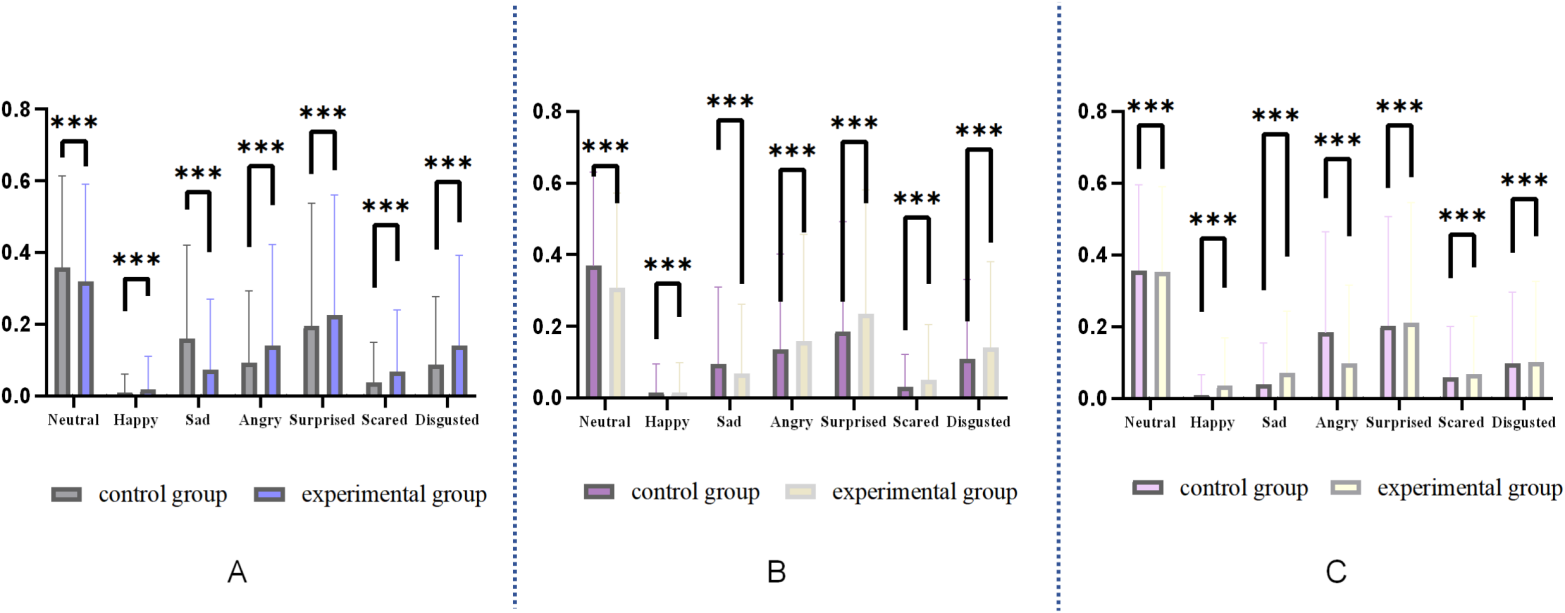
Results from inter‐group facial expression analysis (***p < 0.01). (A) shows significant facial expression discrepancies between the experimental and control groups in Experiment A (Coffee pot). (B) shows significant facial expression discrepancies between the experimental and control group in Experiment B (Panda). (C) shows significant facial expression discrepancies between the experimental and control groups in Experiment C (Scarf).

Significant differences in emotional changes were observed between the two conditions across the three experiments, reflecting how mixed stimuli affect emotional evaluation of products by the study participants (for details see Figure 5). For example, neutral emotion values were generally higher in control conditions than those of the corresponding experimental conditions. Meanwhile, for the other emotional states, like Happy, Sad and Angry, there were also distinct differences in response intensity and dispersion between the control and experimental conditions. Mixed stimuli appeared to exert a significant influence on the patterns and intensities of individual emotional responses.

**FIGURE 6:**
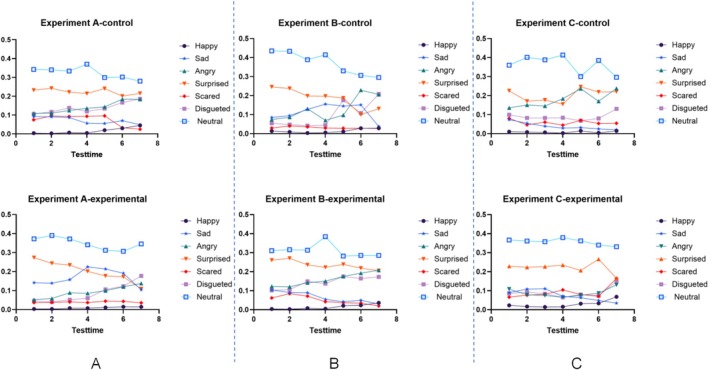
Dynamic changes in facial expressions among the groups over seven test times. (A) represents the changing trends of facial expressions between the experimental and control groups in Experiment A (Coffee pot) at different test time points. (B) represents the changing trends of facial expressions between the experimental and control groups in Experiment B (Panda) at different test time points. (C) represents the changing trends of facial expressions between the experimental and control groups in Experiment C (Scarf) at different test time points.

Specifically, participants in the control conditions displayed low sensitivity and stability and were not excessively disturbed by the external stimulation. In contrast, the experimental conditions were more sensitive and prone to fluctuations, especially reacting strongly in specific scenarios, such as test time 4, during which participants were viewing images. In addition, the degree and complexity of emotional fluctuations were different across the experimental conditions. In the Experiment A (Coffee pot), the experimental group exhibited the most significant emotional fluctuations. During the later experimental phase, negative emotions such as anger and disgust presented with rapid growth (Figure 6A). In the Experiment B (Panda), the experimental group displayed consistently low and stable positive emotions, while negative emotions remained persistently intense (Figure 6B). In the Experiment C (Scarf), the experimental group showed the most negative emotional responses overall, with no observable positive emotional responses observed throughout the session. These negative emotions—sadness, anger and fear—all exhibited sharp peak surges followed by rapid dissipation patterns (Figure 6C).

### 3.7 Vital Signs

**FIGURE 7:**
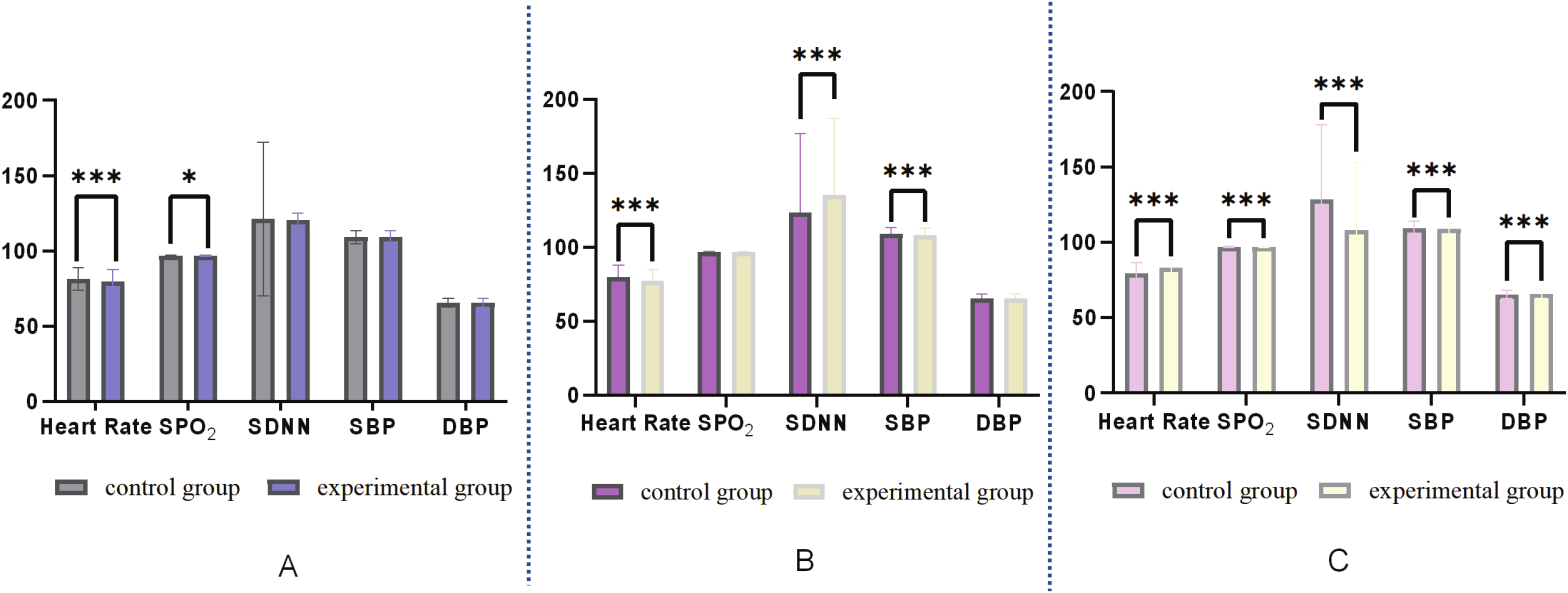
Results from inter‐group analyses of vital signs (*p < 0.05, ***p < 0.01). (A) shows significant intergroup differences in Heartrate and SPO_2_ in Experiment A (Coffee pot). (B) shows significant intergroup differences in Heart rate, SBP and SDNN in Experiment B (Panda). (C) shows significant intergroup differences in all vital sign indicators in Experiment C (Scarf).

Significant differences were found with respect to vital signs between the control and experimental groups across the three experiments (for details see Table S5). Based on inter‐group analyses, Experiment A (Coffee pot) only exhibited significant changes in heart rate (Figure 7A), whereas Experiment B (Panda) and Experiment C (Scarf) showed progressively broader significant differences across multiple physiological indicators, including heart rate, SDNN, SPO_2_ and blood pressure (Figure 7B,C). This trend suggested that stimulation with mixed cultural images gradually disrupted the conventional stable state of visual cognitive processing and triggered a more systematic stress‐induced physiological regulatory response. Notably, the number of significantly different indicators and the magnitude of differences varied across the three experimental conditions, indicating that the intensity of cognitive conflict may differ depending on the specific stimulus conditions. This pattern confirmed a strength‐dependent relationship between cognitive and physiological responses.

**FIGURE 8:**
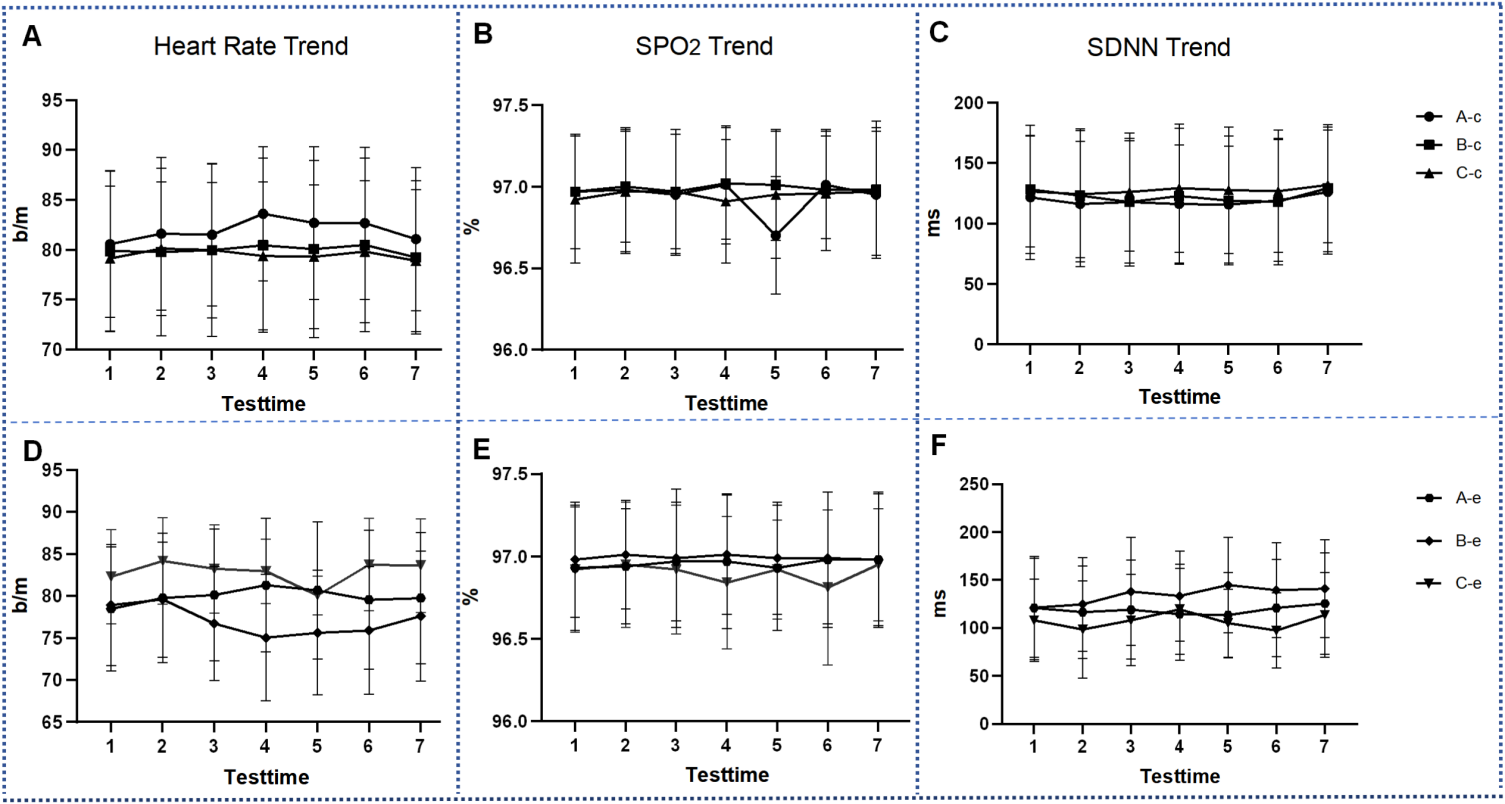
Results from vital signs monitoring. (A) shows the dynamic Heart rate trends of the control group over time across all experiments. (B) shows the temporal SPO_2_ trends of the control group over time across all experiments. (C) shows the dynamic SDNN changes of the control group over time across all experiments. (D) shows the temporal Heart rate trends of the experimental group over time across all experiments. (E) shows dynamic SPO_2_ variations of the experimental group over time across all experiments. (F) shows the temporal changes in SDNN for the experimental group over time across all experiments. A‐c: Experiment A control group; A‐e: Experiment A experimental group; B‐c: Experiment B control group; B‐e: Experiment B experimental group; C‐c: Experiment C control group; C‐e: Experiment C experimental group.

The physiological responses of experimental group remained stable in Experiment A, as reflected in the heart rate (Figure 8A,D), SPO_2_ (Figure 8B,E) and SDNN (Figure 8C,F) trends. While the experimental group in Experiment B exhibited characteristics such as low heart rate (Figure 8A,D), low blood pressure and stable SPO_2_ (Figure 8B,E), accompanied by higher SDNN values (Figure 8C,F), reflecting active parasympathetic nervous activity and strong autonomic regulatory capacity (Shaffer and Ginsberg 2017). Based on these results, participants did not feel conflict but instead demonstrated curiosity and exploratory tendencies, indicating a positive and well‐adapted psychophysiological state. In Experiment C, experimental group presented with an accelerated heart rate (Figure 8D), elevated blood pressure and suppressed respiration, accompanied by a marked reduction in SDNN (Figure 8F), indicating a high level of arousal and a low‐adaptability state, which suggest that the mixed culture stimuli presented to this group evoked strong cognitive conflict, resulting in an aversive physiological experience, more details see Figure 8.

## 4 Discussion

**FIGURE 9:**
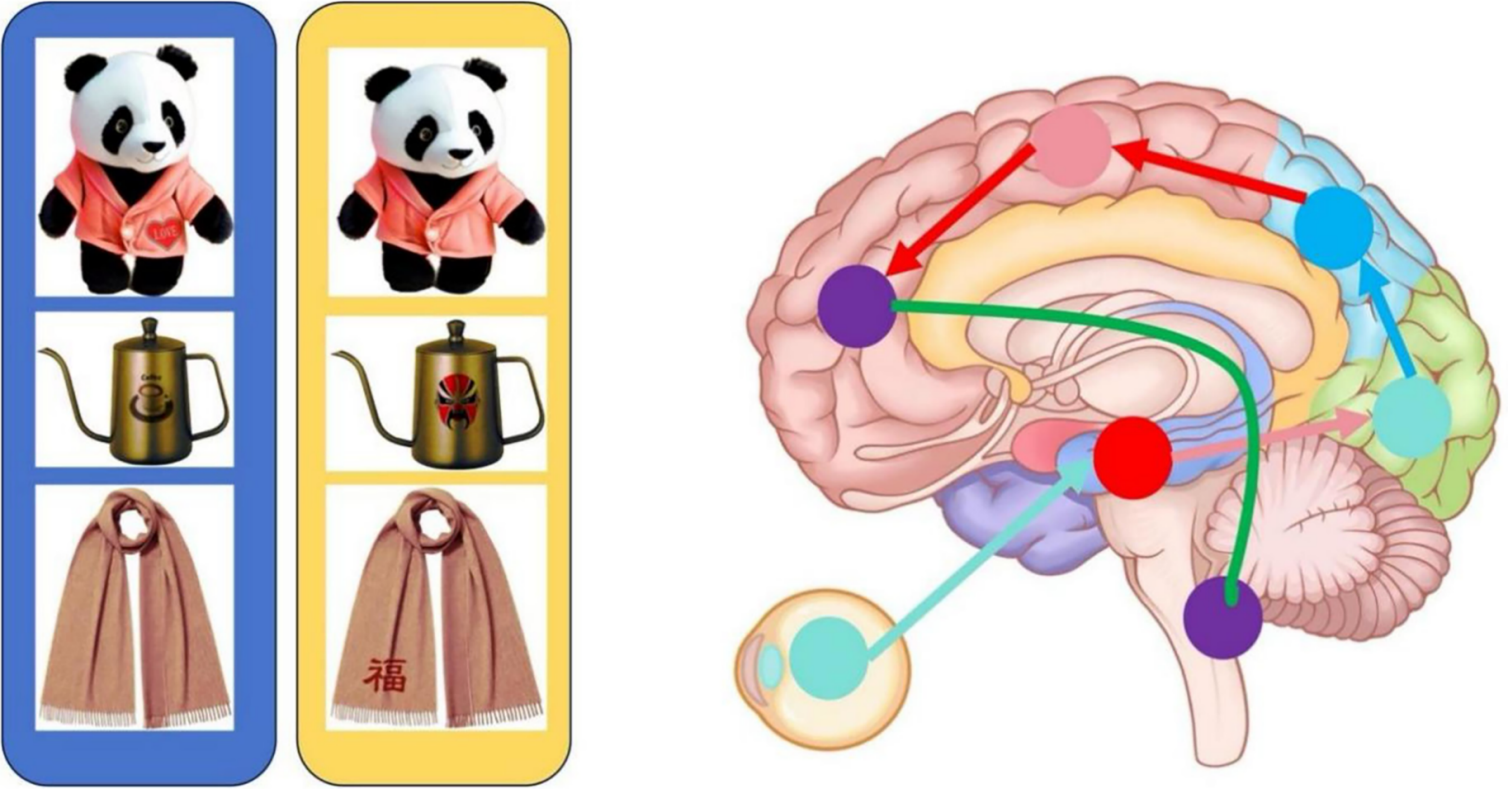
The mechanism by which visual stimulation induces cognitive conflict and influences decision‐making.

The present study employed three independent experiments, each with a control group and an experimental group to systematically investigate the specific effects of mixed culture stimuli on behavioural and neuro‐physiological responses. Results were analysed by integration of multi‐modal data, including fNIRS to measure brain functional connectivity, and changes in facial expressions and vital signs. These data were coupled with subjective evaluations. Importantly, the exploratory analyses suggested an apparent gradient pattern across the three experiments that appeared to align with the behavioural outputs, namely purchase intention. Specifically, Experiment A (Coffee pot) showed no significant differences, Experiment B (Panda) exhibited marginal effects and Experiment C (Scarf) demonstrated the most pronounced differences, including a trend‐level difference in SMG‐R‐FPA functional connectivity. For study participants, exposure to culturally mixed stimuli may induce conflict within their internal cultural cognitive framework. Through multi‐response processes, potentially involving changes in brain functional connectivity, emotional arousal and vital signs, individuals appear to make acceptance or rejection decisions based on their cultural adaptability and the capacity for emotion regulation and subjective valuation (for details see Figure 9). This mechanism is consistent with the research findings of Hua et al. (2019) and Kim and Sasaki (2014), which demonstrate that the degree of cognitive conflict coordination directly influences purchase intention. Both studies highlight the impact of interactions between cultural stimuli and individual cognition on behavioural decision‐making. The gradient pattern across the three experiments suggested that the potential negative effect of mixed culture on purchase intention may not be uniform, but is likely dependent on a threshold of cognitive conflict.

In terms of the strength of functional connectivity in the brain regions of the different groups, significant activation differences were found between the experimental and control groups in several brain regions. We integrate our specific neural findings with established consumer neuroscience frameworks, specifically focusing on emotion regulation failure and affective devaluation (Lerner et al. 2015; Lerner et al. 2004). In the experimental group, different stimuli were associated with conflict perception and significant negative arousal, which overloaded the frontoparietal emotion regulation network. Specifically, the functional connectivities between the FPA and the supramarginal gyrus indicate a breakdown in the neural network responsible for linking self‐identity with product valuation. Rather than simply suppressing traditional semantic processing pathways, this decoupling reflects a failure of prefrontal‐mediated emotion regulation. Consequently, the neural system prioritises the stress response, effectively down‐regulating the perceived value of the product—a process termed affective devaluation (Ferreira et al. 2019). The control groups viewed conventional pictures, and the brain quickly completed recognition. With preserved frontoparietal connectivity, indicating intact emotion regulation and stable subjective valuation without the need for in‐depth conflict resolution. Interestingly, this finding aligns with and refines the Dual‐Process Theory by specifying the neuro‐valuation mechanisms underlying the cognitive shifts. A previous study used fMRI technology to investigate neural responses when participants were exposed to cultural symbols significantly different from their own cultural background, revealing significantly increased activation in the anterior cingulate cortex (ACC) and further demonstrating that cultural stimuli can elicit changes in neurophysiological states, providing validation from different methodological perspectives for the objective impact of cultural stimuli on brain function (Chiao et al. 2010).

Regarding facial expressions, significant differences were found among these groups, indicating that mixed culture stimulation effectively evoked observable emotional responses in the study participants. The experimental groups appeared to be more prone to negative emotions in the later phase, exhibited a stronger peak in neutral emotions and showed greater overall emotional volatility. Based on the changing trends of facial expressions across the groups, it can be observed that the conflict arising from stimulation with mixed cultural images evoked cognitive confusion and overloaded regulatory processes among participants in the study (Ekman and Friesen 1978), which directly contributed to the affective devaluation observed in the behavioural outcomes. Similarly, early in 2009, a previous study reported a significant increase in negative emotions and greater emotional volatility, in agreement with the emotional reactions observed in the experimental group under mixed cultural stimuli. This convergence of results across different study subjects and measurement methods validates the universality of the impact of mixed cultural stimuli on emotional responses.

Cognitive conflicts may be triggered by different stimuli and can lead to corresponding physiological adjustments, as has been demonstrated in other studies (Botvinick et al. 2001; Critchley 2002). In support of this, in the present study, the vital signs of the control groups remained stable, while the experimental groups presented with significant vital sign differences. The control groups were found to have intermediate levels for each measured vital sign, with moderate fluctuations, demonstrating relative stability. Differences within the control groups were minimal, with heart rate curves showing a high degree of overlap, indicating that different types of conventional stimulation did not evoke significant differences in physiological responses, and confirming the validity and stability of the control groups as an experimental control. In contrast, the experimental groups displayed significantly stronger responses, which evolved from significant changes in single indicators to comprehensive activation of multiple metrics. Taken together, our results demonstrate that mixed culture images served as the core stimulus responsible for inducing these physiological differences, with the number and significance of various indicators among the groups effectively reflecting the degree of cognitive conflict intensity that drove the emotion regulation failure.

The present study suggested that the intensity of an individual's cognitive conflict correlates with the specific attributes of the stimuli, following exposure to culturally mixed visual stimuli, which is further associated with brain functional connectivity, multi‐dimensional physiological responses and observable differences in facial expressions. These changes may reflect a multi‐level pathway from cognition to behaviour. The experimental results shown in the present study indicate that an individual's decisions as a consumer appear to depend on whether the stimuli content exceeds their internal acceptance threshold. These findings not only provide exploratory evidence for classic theories, namely, the cognitive dissonance theory and the appraisal theory of emotion, but also suggest affective devaluation as a potential process linking cognitive conflict to consumer rejection, highlighting the acceptance threshold as a key psychological variable in predicting consumer decision making toward mixed culture stimuli.

### 4.1 Limitations

The present study only recruited participants from among college students, with a relatively narrow age range of 18–22 years. This study sample may differ systematically from the broader population in terms of cognitive style, cultural exposure and purchasing ability. Future studies can recruit more participants from a wider range of ages and occupational backgrounds to improve the generalisability and ecological validity of the results. Furthermore, the effect of individual differences in acceptance thresholds can be further explored to provide theoretical support and practical guidance for consumer positioning in cross‐cultural marketing strategies.

## Author Contributions

**Yutong Ma:** project administration, writing – review and editing, data curation. **Haiqi Xiang:** software, formal analysis. **Shuang Wei:** visualization. **Fangfang Liu:** supervision, visualization. **Peiyao Liu:** visualization. **Xiangyu Yan:** software, investigation. **Fan Xu:** funding acquisition, resources, writing – review and editing. **Wenhao Lv:** methodology, validation. **Yelan Zhang:** investigation. **Caiqing Chen:** investigation. **Yinfeng Tan:** formal analysis. **Yanping He:** data curation, formal analysis, writing – original draft. **Lu Gan:** investigation. **Hong Shi:** conceptualization, supervision, methodology.

## Funding

This study was supported by the Southwest Minzu University Research Startup Funds：RQD2021038, the National Key R&D Program of China NO. 2023YFE0108400, Key Discipline Project at the School of Public Health, Chengdu Medical College (No. 21), School joint funding: 23LHPDZYB08, Sichuan applied psychology research center, CSXL‐24215.

## Ethics Statement

This study was previously approved by an institutional ethics committee (2023NO.113).

## Consent

The participants provided their written informed consent to participate in this study.

## Conflicts of Interest

The authors declare no conflicts of interest.

## Supporting information


**Table S1:** The results of stimuli validation.
**Table S2:** The Relationship between channels' location and brain region.
**Table S3:** Independent Samples *t*‐test Results for Purchase Intention.
**Table S4:** Complete functional connectivity results for all a priori defined ROI pairs across three experiments.
**Table S5:** The vital signs difference between different group (mean [sd]).

## Data Availability

The data that support the findings of this study are available from the corresponding author upon reasonable request.
