# Supplementary material for: The Effect of Culture Mix or Not Conflict Against the Decision Making—A fNIRS Study
Source: Int J Psychol. 2026 Jul 24;61(5):e70253. doi: 10.1002/ijop.70253 (PMC13400748; doi:10.1002/ijop.70253)
Supplement: Supplementary file 1 — Table S1: The results of stimuli validation. Table S2: The Relationship between channels' location and brain region. Table S3: Independent Samples t‐test Results for Purchase Intention. Table S4: Complete functional connectivity results for all a priori defined ROI pairs across three experiments. Table S5: The vital signs difference between different group (mean [sd]). [file IJOP-61-e70253-s001.docx]

**Supplementary Table 1.The results of stimuli validation**

| **Feature** | **Mean**  **（control gruops）** | | **M**  **(experimental groups）** | **t** | **p** |
| --- | --- | --- | --- | --- | --- |
| brightness | 189.342 | 189.357 | | -0.002 | 0.999 |
| saturation | 56.721 | 56.73 | | -0.001 | 0.999 |
| complexity | 21.021 | 24.482 | | -0.909 | 0.426 |
| entropy | 5.231 | 5.301 | | -0.137 | 0.898 |

**Supplementary Table 2. The Relationship between channels’ location and brain region.**

| **Channel** | **MIN** | | | **Anatomic partition** | **Percentage of Overlap %** |
| --- | --- | --- | --- | --- | --- |
| CH01 | 64.000 | -11.333 | 43.667 | 6 - Pre-Motor and Supplementary Motor Cortex | 0.727 |
| CH02 | 55.333 | -10.667 | 55.667 | 6 - Pre-Motor and Supplementary Motor Cortex | 0.375 |
| CH03 | 59.000 | 32.333 | 2.667 | 47 - Inferior prefrontal gyrus | 0.481 |
| CH04 | 63.000 | 16.333 | 18.333 | 45 - pars triangularis Broca's area | 0.474 |
| CH05 | 47.000 | 56.000 | 3.000 | 10 - Frontopolar area | 0.831 |
| CH06 | 18.667 | 73.000 | 1.667 | 10 - Frontopolar area | 1.00 |
| CH07 | 32.667 | 64.333 | 17.333 | 10 - Frontopolar area | 1.00 |
| CH08 | -12.667 | 74.000 | 0.667 | 10 - Frontopolar area | 0.960 |
| CH09 | -42.333 | 58.667 | 0.333 | 10 - Frontopolar area | 0.973 |
| CH10 | -27.333 | 65.667 | 16.667 | 10 - Frontopolar area | 1.00 |
| CH11 | -55.000 | 37.333 | -1.333 | 47 - Inferior prefrontal gyrus | 0.702 |
| CH12 | -59.000 | 22.000 | 15.000 | 45 - pars triangularis Broca's area | 0.692 |
| CH13 | -42.667 | -1.667 | 61.333 | 6 - Pre-Motor and Supplementary Motor Cortex | 0.996 |
| CH14 | -29.667 | -2.667 | 68.333 | 6 - Pre-Motor and Supplementary Motor Cortex | 1.000 |
| CH15 | 45.000 | -28.000 | 68.000 | 3 - Primary Somatosensory Cortex | 0.368 |
| CH16 | 33.333 | -27.667 | 74.000 | 3 - Primary Somatosensory Cortex | 0.227 |
| CH17 | 52.667 | 43.000 | 17.000 | 46 - Dorsolateral prefrontal cortex | 0.846 |
| CH18 | 54.667 | 28.667 | 32.333 | 9 - Dorsolateral prefrontal cortex | 0.484 |
| CH19 | 40.333 | 48.333 | 32.333 | 9 - Dorsolateral prefrontal cortex | 0.509 |
| CH20 | 4.333 | 69.333 | 17.667 | 10 - Frontopolar area | 1.00 |
| CH21 | 15.667 | 63.000 | 33.667 | 9 - Dorsolateral prefrontal cortex | 0.454 |
| CH22 | -12.333 | 62.333 | 34.333 | 9 - Dorsolateral prefrontal cortex | 0.502 |
| CH23 | -48.333 | 46.667 | 14.333 | 46 - Dorsolateral prefrontal cortex | 0.765 |
| CH24 | -36.667 | 50.667 | 30.667 | 9 - Dorsolateral prefrontal cortex | 0.388 |
| CH25 | -50.667 | 32.333 | 29.667 | 46 - Dorsolateral prefrontal cortex | 0.766 |
| CH26 | -61.333 | -5.667 | 40.667 | 6 - Pre-Motor and Supplementary Motor Cortex | 0.979 |
| CH27 | -52.667 | -4.333 | 54.333 | 6 - Pre-Motor and Supplementary Motor Cortex | 0.753 |
| CH28 | 45.333 | -7.667 | 63.667 | 6 - Pre-Motor and Supplementary Motor Cortex | 0.874 |
| CH29 | 32.333 | -7.333 | 69.667 | 6 - Pre-Motor and Supplementary Motor Cortex | 1.00 |
| CH30 | -45.000 | -26.000 | 67.000 | 3 - Primary Somatosensory Cortex | 0.425 |
| CH31 | -33.333 | -24.000 | 73.667 | 6 - Pre-Motor and Supplementary Motor Cortex | 0.461 |
| CH32 | -64.333 | -27.667 | 44.667 | 2 - Primary Somatosensory Cortex | 0.274 |
| CH33 | -54.333 | -24.667 | 58.667 | 2 - Primary Somatosensory Cortex | 0.403 |
| CH34 | 66.000 | -32.333 | 47.333 | 40 - Supramarginal gyrus part of Wernicke's area | 0.647 |
| CH35 | 55.333 | -29.667 | 57.667 | 40 - Supramarginal gyrus part of Wernicke's area | 0.428 |
| CH36 | 35.333 | -88.000 | 31.667 | 19 - V3 | 1.000 |
| CH37 | 24.667 | -102.000 | 15.667 | 18 - Visual Association Cortex (V2) | 0.601 |
| CH38 | 15.667 | -107.000 | 2.333 | 18 - Visual Association Cortex (V2) | 0.993 |
| CH39 | -37.667 | -87.667 | 32.333 | 19 - V3 | 0.977 |
| CH40 | -26.667 | -100.333 | 15.333 | 19 - V3 | 0.584 |
| CH41 | -15.667 | -108.000 | 0.667 | 18 - Visual Association Cortex (V2) | 0.984 |
| CH42 | 20.333 | -26.667 | 77.000 | 6 - Pre-Motor and Supplementary Motor Cortex | 0.387 |
| CH43 | -18.333 | -3.333 | 74.667 | 6 - Pre-Motor and Supplementary Motor Cortex | 1.00 |
| CH44 | -19.333 | -22.667 | 77.000 | 6 - Pre-Motor and Supplementary Motor Cortex | 0.575 |
| CH45 | 20.667 | -4.667 | 75.000 | 6 - Pre-Motor and Supplementary Motor Cortex | 1.00 |
| CH46 | 14.333 | -96.333 | 30.667 | 19 - V3 | 0.997 |
| CH47 | -14.667 | -97.000 | 30.667 | 19 - V3 | 0.986 |
| CH48 | -4.333 | -105.000 | 14.667 | 18 - Visual Association Cortex (V2) | 0.989 |

**Supplementary Table 3. Independent Samples t-test Results for Purchase Intention**

| **Comparison** | **df** | **t** | **MS** | **p** |
| --- | --- | --- | --- | --- |
| Group A-c VS Group A-e | 98 | -0.14 | -0.04 | 0.8855 |
| Group B-c VS Group B-e | 98 | 1.66 | 0.48 | 0.099 |
| Group C-c VS Group C-e | 98 | 2.34 | 0.66 | 0.0211 |

*Footnote:A-c:Experiment A control group;A-e:Experiment A experimental group;*

*B-c:Experiment B control group;B-e:Experiment B experimental group;*

*C-c:Experiment C control group;C-e:Experiment C experimental group;*

**Supplementary Table 4. Complete functional connectivity results for all a priori defined ROI pairs across three experiments**

| Experiment | ROI pair | Group | Mean±SD | T | P |
| --- | --- | --- | --- | --- | --- |
| A | SMG-R-DLPFC | A-c | 0.436 ± 0.228 | 0.74 | 0.459 |
|  |  | A-e | 0.401±0.239 |  |  |
|  | SMG-R-FPA | A-c | 0.438±0.232 | 0.25 | 0.802 |
|  |  | A-e | 0.426±0.257 |  |  |
|  | DLPFC-FPA | A-c | 0.544±0.0186 | 1.06 | 0.294 |
|  |  | A-e | 0.499±0.236 |  |  |
| B | SMG-R-DLPFC | B-c | 0.294±0.271 | -1.38 | 0.170 |
|  |  | B-e | 0.362 ± 0.224 |  |  |
|  | SMG-R-FPA | B-c | 0.349±0.271 | -1.23 | 0.223 |
|  |  | B-e | 0.412±0.243 |  |  |
|  | DLPFC-FPA | B-c | 0.441±0.204 | -2.00 | 0.049 |
|  |  | B-e | 0.516±0.172 |  |  |
| C | SMG-R-DLPFC | C-c | 0.320 ± 0.291 | -1.64 | 0.105 |
|  |  | C-e | 0.413 ± 0.275 |  |  |
|  | SMG-R-FPA | C-c | 0.300 ± 0.326 | -2.21 | 0.030 |
|  |  | C-e | 0.436 ± 0.289 |  |  |
|  | DLPFC-FPA | C-c | 0.504 ± 0.232 | -0.62 | 0.537 |
|  |  | C-e | 0.531 ± 0.202 |  |  |

*Footnote:A-c:Experiment A control group;A-e:Experiment A experimental group;*

*B-c:Experiment B control group;B-e:Experiment B experimental group;*

*C-c:Experiment C control group;C-e:Experiment C experimental group;*

**Supplementary Table 5. The vital signs difference between different group (mean(sd))**

| **Group** | **A-c** | **B-c** | **C-c** | **A-e** | **B-e** | **C-e** | **Total** | **Test** |
| --- | --- | --- | --- | --- | --- | --- | --- | --- |
| N | 16,978 0.218 | 18,158 0.234 | 17,237 0.222 | 15,646 0.201 | 7,076 0.091 | 2,631 0.034 | 77,726 1.000 |  |
| Heart Rate | 81.528 (7.387) | 79.739 (8.230) | 79.350 (7.274) | 79.828 (7.861) | 77.241 (7.482) | 83.477 (5.199) | 79.962 (7.725) | <0.001 |
| SPO2 | 96.969 (0.371) | 96.985 (0.375) | 96.915 (2.006) | 96.961 (0.391) | 96.989 (0.379) | 96.916 (0.378) | 96.959 (0.998) | <0.001 |
| SDNN | 121.239 (51.028) | 123.538 (53.444) | 128.358 (49.646) | 120.681 (51.354) | 135.426 (51.512) | 108.116 (45.029) | 124.078 (51.504) | <0.001 |
| SBP | 109.233 (4.309) | 109.142 (4.329) | 109.470 (4.907) | 109.241 (4.347) | 108.328 (4.402) | 108.876 (3.777) | 109.170 (4.461) | <0.001 |
| DBP | 65.535 (2.818) | 65.602 (2.826) | 65.456 (3.057) | 65.584 (2.818) | 65.661 (2.899) | 65.790 (2.796) | 65.564 (2.882) | <0.001 |
| p<0.05 means significant | | | | | | | | |

*Footnote： A-c:**Experiment A control group;A-e:Experiment A experimental group;*

*B-c:Experiment B control group;B-e:Experiment B experimental group;*

*C-c:Experiment C control group;C-e:Experiment C experimental group;*
